# Expression analysis of carbohydrate antigens in ductal carcinoma *in situ *of the breast by lectin histochemistry

**DOI:** 10.1186/1471-2407-8-136

**Published:** 2008-05-14

**Authors:** Soheila Korourian, Eric Siegel, Thomas Kieber-Emmons, Behjatolah Monzavi-Karbassi

**Affiliations:** 1 Winthrop P. Rockefeller Cancer Institute, University of Arkansas for Medical Sciences, Little Rock, AR, USA; 2 Department of Pathology, University of Arkansas for Medical Sciences, Little Rock, AR, USA; 3 Department of Biostatistics, University of Arkansas for Medical Sciences, Little Rock, AR, USA

## Abstract

**Background:**

The number of breast cancer patients diagnosed with ductal carcinoma *in situ *(DCIS) continues to grow. Laboratory and clinical data indicate that DCIS can progress to invasive disease. Carbohydrate-mediated cell-cell adhesion and tumor-stroma interaction play crucial roles in tumorigenesis and tumor aggressive behavior. Breast carcinogenesis may reflect quantitative as well as qualitative changes in oligosaccharide expression, which may provide a useful tool for early detection of breast cancer. Because tumor-associated carbohydrate antigens (TACA) are implicated in tumor invasion and metastasis, the purpose of this study was to assess the expression of selected TACA by lectin histochemistry on DCIS specimens from the archival breast cancer tissue array bank of the University of Arkansas for Medical Sciences.

**Methods:**

For detection of TACA expression, specimens were stained with *Griffonia simplicifolia *lectin-I (GS-I) and *Vicia vilosa *agglutinin (VVA). We studied associations of lectin reactivity with established prognostic factors, such as tumor size, tumor nuclear grade, and expression of Her-2/neu, p53 mutant and estrogen and progesterone receptors.

**Results:**

We observed that both lectins showed significant associations with nuclear grade of DCIS. DCIS specimens with nuclear grades II and III showed significantly more intense reactivity than DCIS cases with nuclear grade I to GS-1 (Mean-score chi-square = 17.60, DF = 2; *P *= 0.0002) and VVA (Mean-score chi-square = 15.72, DF = 2; *P *= 0.0004).

**Conclusion:**

The results suggest that the expression of VVA- and GS-I-reactive carbohydrate antigens may contribute to forming higher grade DCIS and increase the recurrence risk.

## Background

Breast carcinoma is the most common malignancy and currently the second leading cause of cancer death in women in the United States. An increasing number of women choose more sensitive screening with digital mammograms and magnetic resonance imaging, which has ultimately resulted in dramatic increase in the diagnosis of ductal carcinoma *in situ *(DCIS) during recent years [1-4]. The majority of invasive breast cancers likely develop over extended periods of time from pre-invasive lesions such as DCIS and culminating in metastatic disease [5-9]. Untreated DCIS lesions develop into invasive breast cancer with an average progression rate of 43% as estimated by using the results of 8 different independent studies performed with a wide range of follow-up time [3].

An increased risk of a recurrence as DCIS or invasive cancer was associated with initial DCIS lesions that were larger than 10 mm or were of high or intermediate nuclear grade [10-14]. High nuclear grade of initial lesions and cancer recurrence were significantly associated with increased rates of metastasis and breast cancer death [15,16]. Therefore, nuclear grade is used as a major determinant of therapy and medical treatment decision for DCIS patients [11,17,18].

Tumor initiation and growth is associated with modifications to the structure of glycan residues belonging to glycoproteins, glycolipids and proteoglycans present at the cell surface [19]. Aberrant glycosylation, arising from dysfunction of glycosyltransferases and/or glycosidases, most often results in a shortening of the glycan chains or an over-expression of structures on cells that are normally absent or scarce. It is well-recognized that aberrant carbohydrate expression is relevant to tumor metastasis and poor prognosis for cancer patients [20-22].

A feature of adenocarcinoma cells is their high accumulation of an abnormal mucin carbohydrate-substitution pattern compared with mucin of normal epithelial cells. This phenomenon is observed in most types of human cancers including breast cancer [23-26]. The accumulation of carbohydrate structures reactive with *Vicia vilosa *agglutinin (VVA) and *Griffonia simplicifolia *lectin-I (GS-I) is closely associated with development, progression, and metastasis of breast adenocarcinoma [27-30]. VVA reacts with N-acetyl-galactosamine (GalNAc) residues of glycoproteins that are predominantly expressed on the mucin MUC1 [29,31]. The expression of VVA-reactive epitopes has been linked to lymphatic invasion and lymph node metastasis in invasive breast cancer [30]. GS-I lectin is a mixture of isolectins that bind to Galα1-3Gal and α-GalNAc terminal groups, which are involved in the formation of metastases in murine and human breast carcinomas [32-35].

Because the expression of cell surface carbohydrate structures reactive with VVA and GS-I lectins has been linked to invasive breast cancer and its metastatic potential, we hypothesized that the expression of TACA reactive with these two lectins may define an aggressive phenotype in DCIS. Therefore, the aim of this study was to assess the expression of breast-cancer-related TACA markers reactive with GS-I and VVA lectins in DCIS cases in order to evaluate their presence and determine their correlations with established tumor prognostic factors.

## Methods

### Study design

This retrospective study was conducted on archival tissue-array blocks from patients diagnosed with DCIS between 1990–2002 at the University of Arkansas for Medical Sciences. This study was approved by the University of Arkansas for Medical Sciences Institutional Review Board.

Follow up information was available on all patients ranging from 5 to 16 years. Patients' age, race and tumor characteristics including tumor nuclear grade and size were obtained from the pathology data base whenever possible. For the majority of cases immunohistochemistry results of the molecular markers Her-2/neu, p53 and the estrogen and progesterone receptors were also available in pathology files. For the majority of the cases, immunohistochemistry results of the molecular markers Her-2/neu, p53 and the estrogen and progesterone receptors were also available in the pathology files. Immunohistochemical studies for estrogen, progesterone and p53 were performed on the original blocks using antibodies from Ventana Medical Systems Inc. (Tucson, AZ). Anti ER clone 6F1, anti PR clone1A6 and anti-p53 clone Bp53-11 were used. Any tumor with greater than 10% positivity with estrogen and progesterone were recorded as positive. Any nuclear positivity for p53 was reported as positive. Benign breast tissue within the submitted sections was used as internal positive control according to the pathology protocols. Hercept test by Dako (Carpinteria, CA) was used for Her2-neu testing. The cases were scored according to the DAKO standards of reporting for this antibody. The tumor was reported as negative, if the tumor cells showed no or weak incomplete cytoplasmic membrane staining. They were reported as positive 2+, if the neoplastic cells showed complete cytoplasmic membrane staining in more than 10% tumor cells at weak or intermediate intensity and positive (3+), if the tumor cells showed complete strong cytoplasmic membrane staining. The pathologist on record (SK) reviewed all samples and verified the presence of DCIS on the core used for the analysis. The study conducted on total of 60 available DCIS cases.

### Lectin histochemistry

Staining of tumor specimen with lectins was performed based on a previously described method [36]. Briefly, formalin-fixed paraffin-embedded tissue were cut at four microns and deparaffinized. Heat-induced epitope retrieval was performed using 10× citrate buffer (Dako) in a Fisher Isotemp water bath at 95–99°C. Slides were cooled down for 20 minutes and rinsed in distilled water. Slides then were treated with PBS containing BSA (bovine serum albumin, 20 mg in 10 ml PBS) for 20 minutes. Slides were washed in PBS with added Calcium Chloride (0.06 g to 1000 ml PBS) for 10 minutes with agitation. Sections were then incubated with lectins (2 μg/ml, Vector Laboratories, Burlingame, CA) for 30 min in PBS + 0.2% BSA at room temperature (RT) and washed in PBS. Sections were then incubated with streptavidin-HRP (Vector Laboratories) for 15 min at RT followed by PBS wash. Sections were incubated with diaminobenzidine solution (DAB) for 5 min at RT, washed with distilled water, counterstained with hematoxylin, mounted, and examined under a light microscope. Cytospin slides of VVA and GS-I-positive breast cancer cell line (MDA-MB-231) were used for positive control. Stromal cells of the breast parenchyma did not show any positivity with either lectin. These types of cells were present in all cores and were used as negative internal control.

### ELISA

ELISA was performed as described elsewhere [37]. Briefly, plates were coated with 4 μg/ml of carbohydrate probes attached to a polyacrylamide polymer (PAA) (GlycoTech Corporation, Rockville, MA) in carbonate-bicarbonate buffer at 4°C overnight. Biotinylated lectins were then added and incubated for 1 hour at 37°C, and binding was visualized with streptavidin-HRP (Sigma, St Louis, MO). Absorbance was read, using a Bio-Tek ELISA reader (Bio-Tek instruments, Inc, Highland Park, Vermont).

### Scoring and statistical analysis

Tumor nuclear grade was determined using the Van Nuys grading system [17,38]. The expression of carbohydrate antigens was scored using a four-point staining-intensity scale (from 0, negative; to 3+, strongly positive). The size of initial tumor prior to 1999 was estimated based on the available gross description and the archival slides. The size, measured or estimated, was further categorized as 1 (≤ 1), 2 (> 1 and ≤ 2), 3 (> 2 and ≤ 3), 4 (> 3 and ≤ 4) and 5 (> 4). Association of staining intensities of lectins with age, race, tumor size and grade, estrogen receptor (ER), progesterone receptor (PR), p53, and Her-2/neu was characterized using the Spearman coefficient of rank correlation (r_s_). Associations between staining intensities and other ordinal categorical variables were tested for statistical significance using the Mantel-Haenszel (MH) correlation chi-square test [39]. Staining-intensity associations with Age (the only continuous variable) were tested for significance using the *P *values associated with the rank correlations. The significance level for correlation analyses was set at 0.5% alpha to adjust for the multiple comparisons without inflating Type II error. Staining-intensity associations with nuclear grade of DCIS were further charactserized by computing for each grade the average staining-intensity score as follows: Average score = 0 × (proportion negative) + 1 × (proportion 1+) + 2 × (proportion 2+) + 3 × (proportion 3+). Differences in average scores between grades were tested for significance using the extended Mantel-Haenszel mean-scores chi-square test [39]. Recurrence-free survival (RFS) was also examined in grade I DCIS patients. RFS was measured from the year of diagnosis to the year of recurrence, or censored at last follow-up (2007) if no recurrence occurred by the end of the year; the one death in 1999 for unrelated cause was censored at death for competing risks. For association with RFS, staining intensities were dichotomized as negative (0) versus positive (1+ or higher), and assessed for significance using the log-rank test. Statistical analyses were conducted using SAS Version 9.1.3 (The SAS Institute, Cary, NC, USA).

## Results

### Patient population and tumor characteristics

Table 1 summarizes tumor size, nuclear grade, expression levels of ER, PR, p53 and Her-2/neu, and age and race of the breast cancer patients whose tumors were analyzed in the present study. The distribution among subclasses in each category is shown. African American women represented 25% of the patient population. The age of patients diagnosed with DCIS ranged from 40 to 79 years with 58.5 years of age as the median.

**Table 1:** Characteristics of patients and tumors used for this study

Factor	Category	Number	Percentage
Tumor size (cm)	≤ 1	13	21.7
	> 1, ≤ 4	23	38.3
	> 4	11	18.3
	Unknown	13	21.7
Tumor grade	I	14	23.3
	II	23	38.3
	III	16	26.7
	Unknown	7	11.7
ER	Negative	19	31.7
	Positive	37	61.7
	Unknown	4	6.7
PR	Negative	36	60.0
	Positive	21	35.0
	Unknown	3	5.0
Her-2/neu	Negative	31	51.7
	Positive	21	35.0
	Unknown	8	13.3
P53	Negative	41	68.3
	Positive	15	25.0
	Unknown	4	6.7
Race	African American	15	25.0
	Caucasian	44	73.3
	Others	1	1.7
Age	Minimum	40	
	Maximum	79	
	Average	59	
	Median	58.5	

### Specificity of lectins used

**Figure 1:**
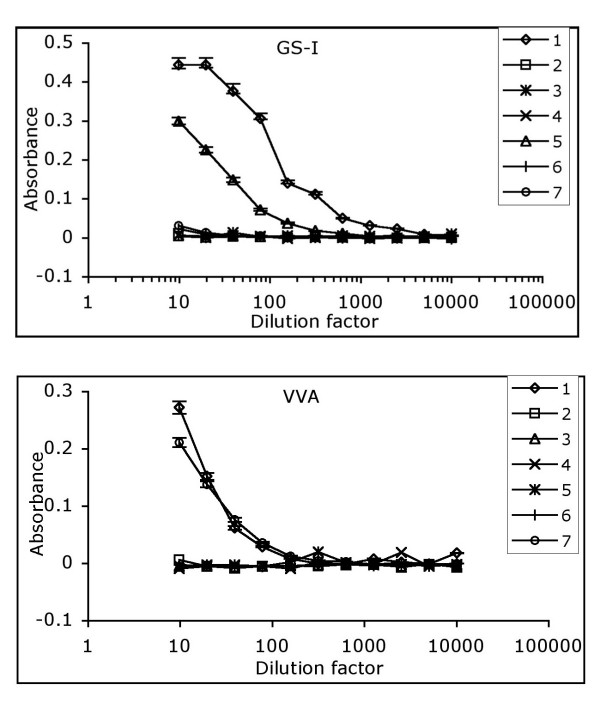
**Carbohydrate specificity of GS-I and VVA lectins.** Reactivity of GS-I and VVA lectins with an array of carbohydrate probes was tested by a standard ELISA. Carbohydrate probes include: 1, GalNAcα 1→3GalNAcα-PAA; 2, Galβ1→4GlcNAcβ-PAA; 3, Galβ1→3GalNAcα-PAA; 4, GlcNAcβ1→3Galβ-PAA; 5, Galα1→3Galβ-PAA; 6, GlcNAcβ1→4GlcNAcβ-PAA; 7, β-GalNAc-PAA. The starting concentration of each lectin was 10 μg/ml that was further serially diluted. The assay was repeated three times with similar results. SD is shown for reactive sugars only.

We examined reactivity of these two lectins against an array of carbohydrates by ELISA to confirm their binding specificity (Figure 1). Both VVA and GS-I bind to terminal α-GalNAc structures. VVA did bind to both α-GalNAc and β-GalNAc but GS-I only reacted with α-GalNAc. GS-I bound to Galα1-3Gal as expected, but it did not recognize structures with terminal β-Gal such as the Thomsen-Friedenreich (TF) antigen (Galβ1-3GalNAc attached to proteins by an α O-serine or O-threonine linkage). Based on this characterization, it appears that VVA binds to terminal α- and β-GalNAc and GS-1 binds to terminal α-Gal- and α-GalNAc-containing structures. These data confirm that the Tn antigen, which resembles the O-glycan structure GalNAc-α 1-O-Ser/Thr can be recognized by both lectins and may serve as an overlap target molecule in staining with these two lectins.

### Expression of VVA and GS-I-reactive epitopes in DCIS

We characterized the associations of GS-I and VVA reactivity with expression levels of ER, PR, p53 mutant, Her-2/neu, and with age, race, tumor size and nuclear grade (Table 2). Total number of specimens and specimens from African American patients included in each analysis is shown (Table 2). Both lectins showed significant associations with nuclear grade of DCIS, with rank correlations (MH *P *values) of 0.50 (0.0002) for GS-I and 0.48 (0.0005) for VVA. At 0.5% alpha, VVA showed a significant correlation with Her2/Neu (r_s _= 0.53; MH *P *= 0.0004), but GS-1 did not (r_s _= 0.34; MH *P *= 0.015). Neither lectin showed significant correlations with tumor size, ER, PR, or p53. Staining intensities for GS-I and VVA were positively associated with each other (r_s _= 0.80; MH *P *< 10^-8^), suggesting that in most cases the two lectins either bind to a single structure, or to structures that somehow are co-expressed.

**Table 2:** Correlation between prognostic markers, race, age, tumor grade and tumor size with GS-I and VVA expression levels IN DCIS patients

	**GS-I**					**VVA**				
	
	*corr*^*a*^	*MH χ2*^*b*^	*MH P*^*b*^	*N*^*c*^*total*	*N*^*c*^*AA*	*corr*^*a*^	*MH χ2*^*b*^	*MH P*^*b*^	*N*^*c*^*total*	*N*^*c*^*AA*
Tumor grade	0.50	14.04	**0.0002**	51	12	0.48	12.31	**0.0005**	51	13
Tumor size	0.04	0.29	0.59	44	9	0.01	0.10	0.76	45	11
ER	-.19	2.22	0.14	52	12	-.26	3.92	0.048	52	14
PR	-.21	3.44	0.064	51	11	-.20	3.80	0.051	50	13
p53	0.29	3.13	0.077	49	12	0.25	2.23	0.14	48	14
Her-2/neu	0.34	5.98	0.015	49	11	0.53	12.75	**0.0004**	48	13
Age	0.05	---	0.72^*d*^	56	12	-.04	---	0.75^*d*^	55	14
Race	0.06	0.08	0.78	55	12	-.16	1.53	0.22	54	14

a: Spearman's coefficient of rank correlation with lectin staining-intensity score.

b: Chi-square statistic and *P *value, Mantel-Haenszel (MH) test for correlation between ordinal categorical variables. *P *values shown in bold face are statistically significant at 0.5% alpha.

c: Number non-missing. Total is total non-missing; AA is non-missing African-Americans.

d: *P *value for rank correlation of Age (continuous variable) with lectin staining-intensity score.

**Figure 2:**
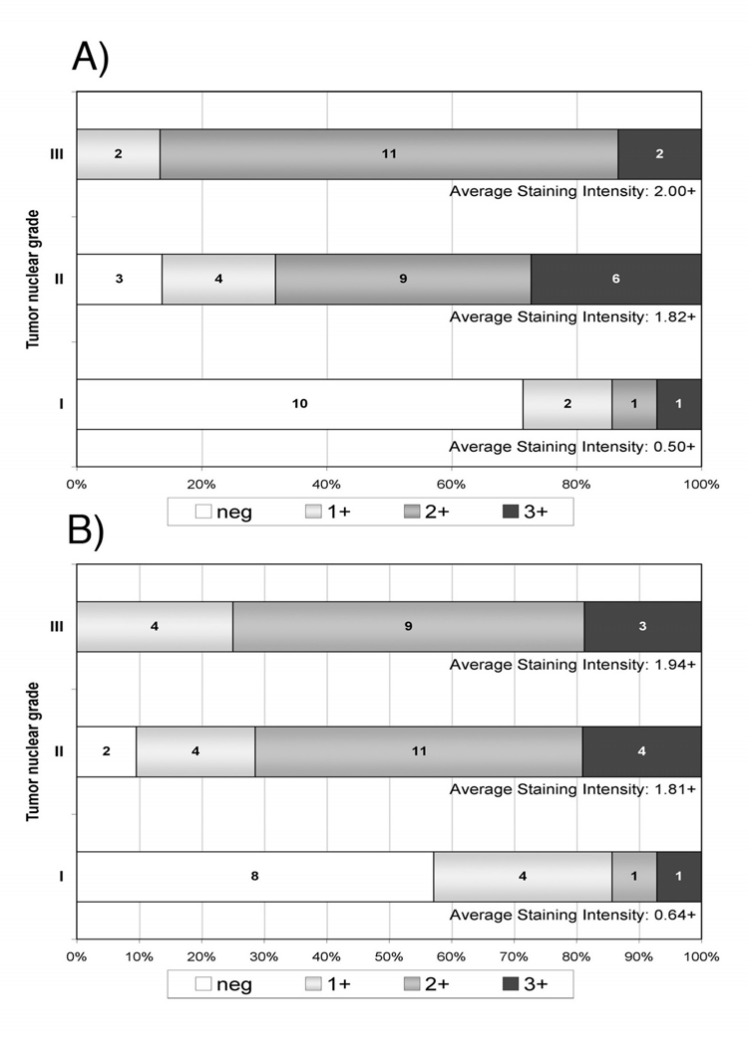
**Distribution of lectin staining intensity among DCIS specimens with low (grade I), intermediate (grade II) and high (grade III) nuclear grade.** Vertical axes show DCIS grade. Numbers inside stacked bars show number of patients with each level of staining intensity while the horizontal axes show the patient numbers as proportions of total patients per grade. For each grade, the average staining-intensity score was calculated as: 0 × (proportion neg) + 1 × (proportion 1+) + 2 × (proportion 2+) + 3 × (proportion 3+). **A) **shows that average staining intensity for *Griffonia simplicifolia *lectin-I (GS-I) was significantly higher in grades II and III than in grade I (Mean-score chi-square = 17.60, DF = 2; *P *= 0.0002). **B) **shows that average staining intensity for *Vicia vilosa *agglutinin (VVA) was significantly higher in grades II and III than in grade I (Mean-score chi-square = 15.72, DF = 2; *P *= 0.0004).

We examined in more detail the nature of the lectin-staining associations with nuclear grade of DCIS (Figure 2). GS-1 showed significantly more intense staining in nuclear grades II and III compared to grade I (Mean-score chi-square = 17.60, DF = 2; *P *= 0.0002), with average staining-intensity scores (percents negatively stained) of 1.82+ (14%) in grade II and 2.00+ (0%) in grade III, compared to 0.50+ (71%) in grade I (Figure 2A). VVA likewise showed significantly more intense staining in nuclear grades II and III compared to grade I (Mean-score chi-square = 15.72, DF = 2; *P *= 0.0004), with average staining-intensity scores (percents negatively stained) of 1.81+ (10%) in grade II and 1.94+ (0%) in grade III, compared to 0.64+ (57%) in grade I (Figure 2B).

**Figure 3:**
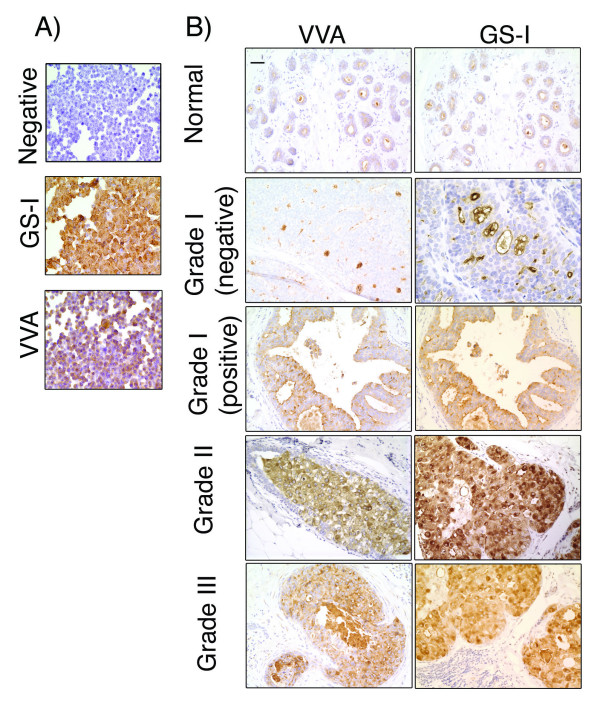
**Lectin histochemical staining of MDA-MB-231 breast cancer cell line and ductal carcinoma in situ.A) **Cytospin slides of MDA-MB-231 cells, which were grown *in vitro *and harvested using enzyme-free buffer. The negative control and staining with GS-I and VVA are shown. MDA-MB-231 breast cancer cell line shows cytoplasmic staining with both lectins and did not show any staining in the absence of the lectins (Negative). 40× Magnification. **B) **Staining of normal breast tissues and tumor samples of different nuclear grade with VVA and GS-I lectins. 20× magnification, bar equals 50 μm.

Results of lectin histochemical staining of a human breast cancer cell line MDA-MB-231 and DCIS specimens are demonstrated (Figure 3). Cytospin slides prepared from MDA-MB-231 human breast cancer cell line were used to set positive controls (Figure 3A). This tumor cell line showed strong positivity with both GS-1 and VVA (Figure 3A). VVA and GS-I showed a similar level of intensity, however a higher percentage of cells showed positivity with GS-1 than with VVA. No reactivity was observed in the absence of either lectin (Negative). We have shown lectin binding to breast cancer cell lines by flow cytometry [36,40,41]. Carbohydrate specificity of VVA and GS-I lectins on paraffin-fixed breast cancer tissues has been shown by others [29,42]. Lectin staining of normal samples and representative DCIS cases with various grades are demonstrated (Figure 3B). Stromal cells show no staining and the benign breast parenchyma shows only apical staining with both GSI and VVL. Grade I DCIS cases, which were scored as negative predominantly showed apical staining with minimal cytoplasmic staining, similar to the pattern and level of staining within benign breast parenchyma. This level of expression is considered negative. In grade I DCIS cases that were scored as positive, we observed a diffuse cytoplasmic staining with both VVL and GS1 clearly above the level of expression in normal breast parenchyma. This pattern of staining was more similar to the staining pattern seen in the high grade DCIS. Grades II and III showed strong diffuse cytoplasmic staining. We also observed that a variation in the level of expression existed within the neoplastic cells in high grade DCIS.

**Figure 4:**
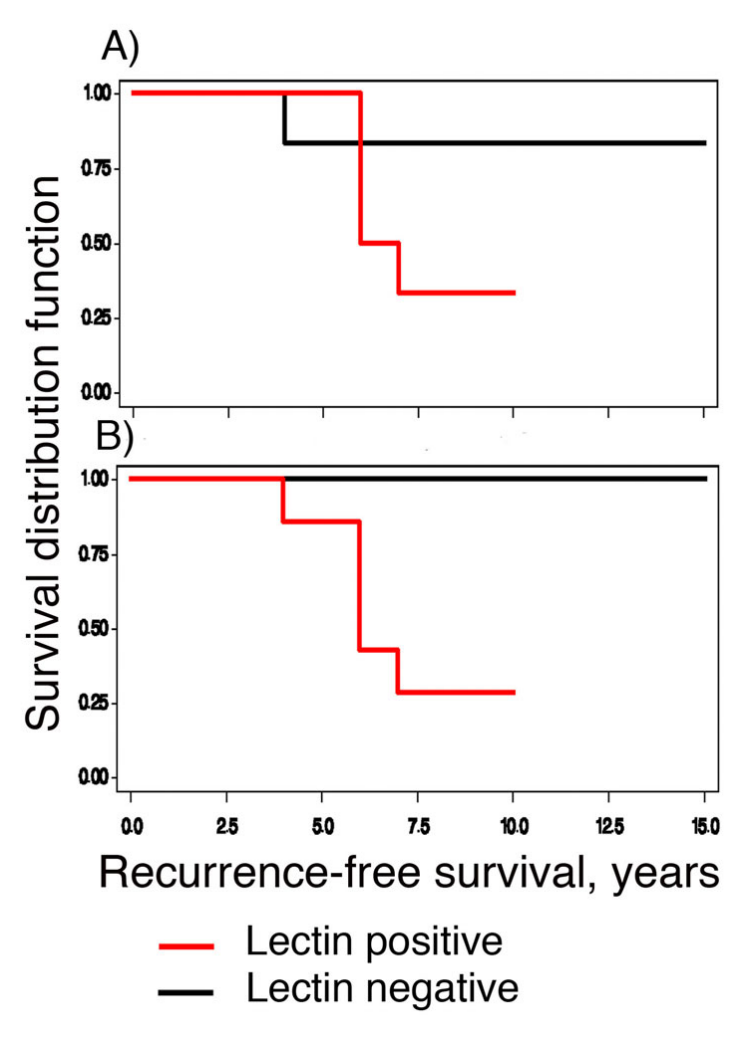
**Kaplan-Meier curves for Recurrence-Free Survival (RFS) in nuclear grade I DCIS, as a function of positive staining intensity.A **shows that 1 of 7 GS-1-negative subjects had a recurrence in their 4th year of follow-up, while 4 of 6 GS-positive subjects had recurrences in their 6th and 7th year of follow-up (log-rank *P *= 0.15). **B **shows that none of the 6 VVA-negative subjects had recurrences during follow-up, whereas 5 of the 7 VVA-positive subjects had recurrences in their 4th, 6th, and 7th year of follow-up (log-rank *P *= 0.02).

Of the fourteen patients with grade-I DCIS, follow-up information was available for 13. Further inspection of the data revealed that five of the 13 showed recurrence in the fourth, sixth, and seventh year of follow-up after their original diagnosis. None of the 13 showed p53 or Her-2-neu amplification, two of 13 were ER-negative and six of 13 were PR-negative. One of the13 patients died in the third year of follow-up of an unrelated cause. The effect of lectin positivity on RFS was examined using survival analysis. Figure 4A shows that 1 of 7 GS-1-negative subjects had a recurrence in her 4th year of follow-up, while 4 of 6 GS-I-positive subjects had recurrences in their 6th and 7th year of follow-up (log-rank *P *= 0.15). Figure 4B shows that none of the 6 VVA-negative subjects had recurrences during follow-up, whereas 5 of the 7 VVA-positive subjects had recurrences in their 4th, 6th, and 7th year of follow-up (log-rank *P *= 0.02). The effect on RFS of negativity for ER or PR was examined, but was not significant (results not shown).

## Discussion

The belief that DCIS is a precursor of invasive breast cancer necessitates the search for finding specific DCIS-associated antigens to identify patients at greater risk for development of invasive cancer and recurrence. Many studies have examined molecular markers in DCIS that are used in clinical practice for invasive breast cancer [43-51]. An overall similarity is reported in the expression of these markers in DCIS and invasive cancer, but with no definite conclusion as to the relevance of these markers to tumor invasion, recurrence and progression.

To search for possible DCIS-associated molecular markers that recognize high-risk DCIS patients, we performed a lectin histochemical staining focusing on breast-cancer-relevant TACAs and evaluated their correlations with tumor prognostic factors. We observed positive correlations between the expression of VVA- and GS-I-reactive structures and tumor grade in DCIS patients. Because tumor grade is the most relevant pathologic determinant of disease with invasive potential, these data suggest that glycoconjugates containing these antigens are associated with invasiveness in DCIS. Tumor grade has been shown to predict prognosis using four gene-expression-based models (70-gene, wound response, two-gene ratio, and recurrence score) [52] and has been used as a major component in prognostic classification of DCIS [11,17,18]. The statistically significant positive correlations observed between the grade of DCIS and the expression of structures reactive with GS-1 and VVA suggest that these reactive antigens may define a phenotype for disease progression or DCIS transformation to invasive disease.

Lack of association of reactivity of either lectin with age and race indicates that reactive structures are independent of race and age. Together, these data suggest that histological evaluation of the expression of VVA- and GS-I-reactive TACAs may serve as phenotypic markers of not only high grade DCIS as defined by nuclear grade, but they may also define DCIS cases which are at high risk for recurrence. In support of this conjecture is the observation that of a total of 13 Low-grade (grade I) DCIS cases in our study, only seven were found positive for VVA lectin and interestingly five of these seven cases were diagnosed with recurrences later in their life. The data suggest that the expression of the lectin-reactive structures in DCIS may play a role in tumor relapse. Further studies targeting low-grade DCIS specimens are needed to make conclusive remarks.

The exact nature of reactive carbohydrates in our staining with VVA and GS-I lectins is not known. Our data confirms the specificity of both lectins. We observed that VVA binds to both α-GalNAc and β-GalNAc, which is consistent with data published by others [30]. Specificity of GS-I to α-GalNAc and α-Gal structures is also reported in the literature [33-35,53,54]. However, the α-Gal epitope is supposed to be absent in humans, because of a lack of alpha1,3-galactosyltransferase activity [55,56]. Therefore, the overall correlative nature, observed in this study, between staining of these two lectins suggests that they both may react with a single epitope on potentially many antigens. Consistently, our data indicate that the Tn antigen (α-GalNAc, an O-linked carbohydrate structure) is an overlapping epitope reactive with these two lectins. However, the fact that, at 0.5% alpha, VVA showed a significant correlation with Her-2/Neu, but GS-I did not, suggests that there may be a distinct nature of reactivity for these two lectins on breast cancer specimens. This data also suggests that Her-2/neu protein might be decorated predominantly with β-GalNAc-containing sugar chains rather than structures that contain α-GalNAc. In this regard, while VVA targets mostly O-linked carbohydrates, binding of GS-I lectin to human breast carcinomas may be related to other unknown antigens. Other investigators also reported the reactivity of α-Gal-specifc GS-I-B4 isolectin with human breast cancer tissues [22,42], which suggests that even if there is no α-Gal expression, another reactive structure should exist on human tumors. Ito and coworkers [42], proposed that poly-N-acetyllactosamines of N-linked glycans contain GS-I-B4 specific epitope on human breast cancer cells. Obviously, the identity of GS-I reactive structures on human breast cancer cells is not clear at the present time and further efforts are needed to identify these antigens.

## Conclusion

Our results suggest that the expression of VVA- and GS-I-reactive carbohydrate antigens may contribute to forming higher grade DCIS and increase the recurrence risk. Such carbohydrates might ultimately serve as viable targets for future development of novel therapeutic strategies aiming at early stage of breast cancer. However, the elucidation of their role in biology and patho-physiology of the disease, which needs further research, precedes such efforts. The data also support the hypothesis of using the expression of these structures as prognostic markers for early stage breast cancer that necessitate conducting further studies on much larger sample size of low-grade DCIS specimens with long follow-up time to establish whether low-grade DCIS with a specific glycomic profile can be subject to relapse more often than other tumors of the same category.

## Competing interests

The authors declare that they have no competing interests.

## Authors' contributions

SK was involved in design of the study, pathological review of specimens, staining and scoring of samples, interpretation of results and drafting the manuscript. ES was involved in statistical analysis and drafting and preparing the manuscript. TKE was involved in design, choosing targets and overall writing and review of the manuscript. BMK was involved in design of the study, data analysis, guiding experimental work and acquiring data, interpretation of results, and drafting and preparing the final manuscript. All authors read and approved the final manuscript.

## Pre-publication history

The pre-publication history for this paper can be accessed here:


